# Effect of changed organisation of nutritional care of Danish medical inpatients

**DOI:** 10.1186/1472-6963-8-168

**Published:** 2008-08-07

**Authors:** Karin O Lassen, Edvin Grinderslev, Ruth Nyholm

**Affiliations:** 1 Department of Development and Training, Copenhagen Hospital Co-operation, Bispebjerg Hospital, Copenhagen, Denmark; 2 Centre for Alternative Social Analyses (CASA), Copenhagen, Denmark

## Abstract

**Background:**

Many patients are undernourished during hospitalisation. The clinical consequences of this include lassitude, an increased risk of complications and prolonged convalescence. The aim of the study is 1) to implement a new organisation with a focus on improving the quality of the nutritional care of medical inpatients at risk of undernutrition, and 2) to investigate the effect of the intervention.

**Methods:**

Social and healthcare assistants are educated to the higher level of nutritional and healthcare assistants to provide nutritional care in daily practice to undernourished medical inpatients. The effect of the intervention is investigated before and five months after the employment of the nutritional and healthcare assistants. Data are obtained from structured interviews with patients and staff, and the amount of ordered and wasted food is recorded.

**Results:**

Patients regard the work of the nutritional and healthcare assistant as very important for their recovery and weight gain: the assistant takes care of the individual patient's nutritional requirements and wishes, and she imparts knowledge to the patient about optimum nutrition. Staff members benefit from the knowledge and dedication of the nutritional and healthcare assistant and from her work; the staff is often too busy with other nursing tasks to make it a priority to ensure that patients who are nibblers get sufficient nutrition. The choices of food from the production kitchen are utilised to a higher degree, and more of the food is eaten by the patients. Before the intervention, a 20% increase in ordered food in relation to the food budget is found. During the intervention a 20% decrease in ordered food in relation to the food budget is found, and food wastage decreases from 55% to 18% owing to the intervention.

**Conclusion:**

The job function of the nutritional and healthcare assistants on the medical wards is of great value to patients, nursing staff members and the production kitchen. The quality of the nutritional care of undernourished patients increases significantly, and a considerable optimisation of resources in the production and ordering of food takes place. Hospitals can benefit from implementation of the present organisational model if they focus on improving the quality of the nutritional care of weak and elderly inpatients and on optimisating the use of resources.

## Background

Insufficient nutritional intake and consequent weight loss during disease remains a serious problem for many elderly inpatients. There is ample reason for countering under nutrition during hospitalisation, among others because weight loss resulting from disease is harmful to all elderly people, whether over- or underweight [1]. Many patients eat and drink insufficiently during hospitalisation, and 30 to 50% of elderly patients are undernourished [2-5], and the protein and energy requirements of most of these patients are not met [3,6-9]. The clinical consequences include lassitude, difficulty in mobilising, prolonged convalescence [3,10-14] and an increased risk of pressure wounds [15], phlebitis and infections [16,17].

Intervention studies have shown that patients' protein and energy intake can be increased significantly through optimisation of the nutritional care using the available hospital food [18-22].

To combat under nutrition and its clinical consequences, it appears relevant to incorporate the nutritional care as a priority area in the daily practice of nursing care. Normally, in Danish hospitals the nursing team is jointly responsible for the patients' nutritional care. Nevertheless, several studies suggest that the nutritional care in daily practice is given a low priority in the nursing care.

Some of the identified factors that inhibit optimal nutritional care at the wards are lack of time and interest in nutrition care among the nursing staff, and the collective nature of the responsibility for the practical implementation of nutritional care. Even if responsibility is collective, only few staff members are actively engaged [6,23-28]. So the question is "How do we in daily practice redress this situation?". An organisational model in which a proffessional is hired exclusively to take care of tasks related to nutrition at the bed wards meets the criteria required to raise nutritional standards at the departmental level; the assistant's time is devoted to provide individual nutritional care to nibblers, she has intimate knowledge of and takes an interest in nutrition, and she has general knowledge of the food range offered by the production kitchen with which she is in close contact [26]. In this study we will focus on these aspects; and the aim is to implement and evaluate an organisational model which has a clear division of responsibilities and competencies in the nutritional care with a view to eliminating factors inhibiting optimal nutritional care.

## Methods

### Setting

Three medical wards specialised in lung, gastric and liver disease, and endocrinology at the Medical Centre at Bispebjerg Hospital (Copenhagen Hospital Co-operation) participate in the study. The number of beds at the wards is 20, 22 and 20, respectively. The monthly bed occupancy rate is 95 to 103%. Patients are hospitalised for eight days on average and are usually admitted acutely (data printout from the Office of Economic Affairs, Bispebjerg Hospital).

The hospital food is produced in the central hospital kitchen and transported daily to the wards. At fixed times on a weekly basis, groceries, dairy products, single-portion frozen meals and the like are delivered when ordered by the wards. Each ward has a kitchen with facilities for limited cooking, like baking or preparation of small dishes.

### Design of the intervention study

The pre-intervention parts of data acquisition include:

1) To interview medical inpatients at nutritional risk about the nutritional care (Patient group A)

2) To interview occupational groups about the nutritional care in daily practise

3) To provide supplementary training to three social and healthcare assistants to upgrade them to nutritional and healthcare assistants

The elements in the intervention at three wards are:

4) The nutritional and healthcare assistant at each ward provides nutritional care to patients who are assessed as being at nutritional risk (Patient group C) and has the responsibility for ordering food, recording food wastage, etc.

Data on the effect of the intervention are obtained by:

5) Interviewing medical inpatients about the nutritional care (Patient group C at the ward receiving nutritional care and patient group B at the ward not receiving nutritional care from the nutritional and healthcare assistant)

6) Interviewing occupational groups about the nutritional care in daily practise in the intervention

7) Investigating the effect on the food supply resources.

At the participating wards, no other organisational changes in the nutritional care are carried out during the intervention period. The patients and staff receive oral information about the investigation, underlining the voluntary and anonymous nature of their participation. The study fulfills the Declaration of Helsinki II. The quantitative methods, use of factual and describing data fall outside the framework of the Local Scientific Ethics Committee. The management at the Medical Centre at Bispebjerg Hospital has approved the study.

### Training programme

The educational background of social and healthcare assistants in Denmark is two years of education and ten months of training with a focus on nursing and care of elderly, ill and handicapped persons in relation to personal care, nursing, cooking, etc. The three participating social and healthcare assistants are chosen because they have much interest in and experience from previous jobs with regard to nursing care of nibblers, elderly and patients with senile dementia and lung disease. In this study the assistants receive supplementary education and training in a one-month training programme specified in Table 1. Different professionals such as clinical dietician, psychologist, nurses and a catering officer serve as teachers in the disciplines related to clinical expertise and organisational pedagogy. A nursing officer has the responsibility for the training in practical skills and organisation at the wards. The senior staff nurses are taught how to fill the function of co-operator for the assistants focusing on the improvement of the nutritional care through behavioural changes [29]. At meetings the nursing officer informe the nursing staff at the wards about the new job function, which is illustrated by a video film [30].

**Table 1:** The training program

Field of expertise	Disciplines
Clinical expertise	Physiology, pathology, vitamins and minerals, product orientation, nutrition and nutritional therapy.
Practical skills	Hygiene, cooking, baking, aesthetic presentation and serving of food, and feeding tube care.
Organisation	Cooperation forms (cross-functional, network, etc.), division of responsibilities in the nutritional care, documentation in the nursing care, use of the information and IT system for the ordering of food and the recording of food wastage.
Pedagogy	Philosophy of man, provision of care, communication, patient's perspective, the art of motivating and providing information, personal appearance, self-insight and being present.

Fields of expertise and disciplines in the in-service training programme for the social and healthcare assistants are described in this table. The four fields of expertise are weighted equally and outside instructors are attached as teachers. In total 90 lessons.

### Patient screening

Using the Nutritional Risk Screening 2002, a registered nurse conducts a nutrition screening of adult patients within 24 hours after admission to assess the patient's nutritional risk [31]. If the registered nurse assesses the patient as being at nutritional risk, nutritional therapy is to be initiated. The following parameters are assessed: the patient's energy and protein requirements, three-day dietary records, the patient's protein and energy intake; and individual nutritional care is planned to meet the patient's nutritional needs [32]. In the intervention the registered nurse has the possibility of delegating the responsibility for the nutritional therapy to the nutritional and healthcare assistant working in day duty. The registered nurses select the patients entering the study. The assistant is professionally trained to work actively with nutritional care and has the necessary time to do so in practice. The patients included are typical for the wards.

### The job function of the nutritional and healthcare assistant

The work of the nutritional and healthcare assistant is a full time job to ensure that the patients who are assessed as being at nutritional risk have their nutritional and fluid requirements met; she is responsible for the following:

- The patient is neat and clean at meal times and receives medication with the meal

- The patient sits or lies in the best possible position when eating the meal

- The patient eats the meal in a comfortable and aesthetic environment and receives the required help to eat the meals

- The patient is informed about nutritional and dietary options available from the production kitchen

- The patient is offered appetising small dishes, in-between meals and home baking, fluid and supplements that meet the individual patient's protein, energy and fluid requirements.

In relation to her colleagues she is responsible for

- Preparing individual meals, in-between meals, home baking etc.

- Planning and assigning priorities to her own work time, ensuring that the nutritional and fluid requirements of undernourished patients are met as far as possible

- Working together with the senior staff nurse to implement the new job function

- Working together with the nursing staff and the kitchen assistant on the tasks related to the nutritional care

- Ordering food from the production kitchen and adjusting the orders on a daily basis according to the patients' requirements and wishes

- Co-operation between the ward and the production kitchen in regard to food service

The nutritional and healthcare assistant and the senior staff nurse are jointly responsible for ensuring that all nursing team members have updated knowledge of nutrition and adhere to the relevant hygiene and nutritional guidelines, cf. the accreditation [32]. The nutritional and healthcare assistant and the nursing staff are jointly responsible for documenting the patient's nutritional status, nutritional and fluid requirements and intake and to keep records of the amount of food ordered and food wastage.

### The effect of the intervention

Before the intervention is started and one month before it is terminated, the investigator assesses the effect on the basis of the data collected from a) structured interviews with 75 patients, b) structured interviews with the ward and production kitchen staff, c) the records of the amounts of food ordered and food waste at the wards (data was obtained from the nutritional and healthcare assistants).

### Evaluation by the patients

#### The patients

Medical patients of all ages participate. The inclusion criteria are defined as: 1) the patient is hospitalised for at least five days, 2) the patient is capable of communicating, and 3) the patient eats and drinks a normal diet. The exclusion criteria are defined as: 1) patients with dementia and 2) patients who are severely mentally or physically impaired. These exclusion criteria are based on ethical considerations. The registrered nurses select the patients who meet the criteria, and the patients are included consecutively. The patients receive oral information about the investigation, underlining the voluntary and anonymous nature of their participation. Verbal consent are given to investigator by the study participants.

#### The questionnaire

The questionnaire is based on a questionnaire developed and used in [26]. The questions are designed to focus on issues believed to be related to the patients' nutritional intake and to reflect their experience with the nutritional care they receive. The questionnaire has been validated through interviews with five medical patients to secure that the questions are unambiguous; that the patients understand the questions and that the order of the questions is correct. The questionnaire is used for the structured interviews of the patients. The patient has the option of elaborating on all answers through comments [33], and the comments are written down word-by-word. All interviews are conducted by the same interviewer.

#### Structured interviews

Before the intervention, a total of 30 patients take part in individual structured interviews (group A). The investigator, a research scientist, contacts the patient who receives oral information about the study, and the voluntary nature of the participation is emphasised. No drop-out analysis is made when patients are selected and contacted. In the interview, the investigator reads the questions aloud to the patient, ticks the appropriate boxes according to the patient's answers and writes down any comments the patient has. After five months' intervention, structured interviews are conducted with 25 patients not receiving nutritional care from the nutritional and healthcare assistant (patient group B), and with 20 patients receiving nutritional care from the nutritional and healthcare assistant (patient group C). The criteria for inclusion are unchanged.

### Analysing data

The distribution of the quantitative data is analysed. Patient comments are printed and analysed as text [34]. The presentation of the quantitative data is supplemented by qualitative data from patient comments.

### Evaluation by the staff

Before and after five months of intervention, the proffesional groups are interviwed in eight focus groups about their work with nutritional care, their responsibilities and their experience in nutritional care as practiced [35]. These groups include senior staff nurses, managers and representatives from the production kitchen, nutritional and healthcare assistants, registered nurses, social and healthcare helpers and assistants. A total of 34 and 30 informants participate before and after five months intervention, respectively. The focus in the interviews concerns to identify where in the system changes related to optimum nutritional care are taking place. The interviews are tape-recorded with the informant's permission. The transcribed interviews are analysed as a text with focus on central and opinion-forming topics relating to the intervention study [35].

### The amount of food ordered

Quantitative data are collected about 1) deliveries from the production kitchen to the participating wards during October and November of the calendar years before and during the intervention, 2) the number of patients and 3) bed days during these periods. For all wards at the Medical Centre, data are collected about food budgets and food consumption in the calendar year before and during the employment of the nutritional and healthcare assistants (data printout from the production kitchen, Bispebjerg Hospital).

The total monthly food supply and daily food supply per patient on the participating wards before and during the intervention are calculated. The corresponding values are calculated for four non-participating medical wards at the Medical Centre at Bispebjerg Hospital.

### The amount of food waste

Before the intervention, the amount of food waste in the food containers is recorded for three randomly chosen days: two investigators do the visual assessment in regard to how large a share of the ordered food is left after the staff has served the patients. During the intervention period, the nutritional and healthcare assistants daily as a part of her job records the following: 1) number and type of delivered portions, 2) visual assessment of the number of left-over portions and 3) where relevant they estimate the number of missing portions. On weekday day duty these recordings are carried out by the nutritional and healthcare assistant. On evening, night and weekend duty they are carried out by the other nursing staff. The collected data do not take into account any plate waste or food eaten by staff members.

#### Data processing

The amount of food waste at breakfast, lunch, supper and "late evening" (meal served at 8 pm) is calculated as percentages. Data from the first 10 weekdays of June, October and December are presented.

## Results

### Evaluation by the patients

#### Patient characteristics

Participants are elderly medical patients with chronic disease. Three out of five are mobility-impaired (Table 2). Typical patient diagnoses include acute or chronic lung disease, infectious disease and metabolic disorders. Five to eight out of ten patients in the three groups have experienced unwanted weight loss recently before admission to hospital. To estimate the unwanted weight loss before admission, the patients are asked to estimate their weight loss and the number of days during which they have experienced a weight loss. The patient-reported average daily weight loss is 160 grams. Although this figure has to be judged with caution, it does give an indication of the level of weight loss in this group of patients.

**Table 2:** Characteristics of patients participating in the questionnaire survey

Variable\Patient group	A n = 30	B n = 25	C n = 20
Sex, female/male	18/12	14/11	10/10
Age, years on average (min.; max.)	70(46;89)	76(55;91)	69(55;87)
Length of stay as of the date of the interview*, days on average	11.6	13.2	13.1
Mobility-impaired patients**, (%)	19 (63)	11 (44)	15 (75)
Patients who believe to know their own body weight, (%)	25 (83)	22 (88)	17 (85)
- Patients who have experienced unwanted weight loss recently, (%)	18 (60)	12 (48)	16 (80)
- Patients who remember having experienced weight loss over a period of time, (%)	12 (40)	5 (20)	12 (60)
- Average weight loss according to the patients' own assessments, grams/day***	197	168	134
Patients who have spoken with other staff members about weight loss, (%)	11 (61)	5 (42)	6 (38)

* The total length of stay is not known, as the patients were interviewed during their stay.

** Patients who are bedfast, confined to a wheelchair or walking-impaired.

***If the patient has had unwanted weight loss, the patient is asked how many kilograms over the number of days.

Before and during the intervention the following patients participate; Group A: patients who are admitted before the nutritional and health assistant is employed on the ward. Group B: patients who are admitted during the employment period of the nutritional and healthcare assistant but not receiving her nutritional care. Group C: patients who are receiving nutritional care from the nutritional and healthcare assistant during their entire or part of their stay at the hospital.

#### The patient's dialogue with staff

The results from the structured interviews with the patients show that during their hospitalisation, one to two thirds of the patients who do not receive nutritional care from the nutritional and healthcare assistants (patient groups A and B) have spoken with other staff members about weight loss (Table 2). Patients receiving nutritional care from the nutritional and healthcare assistants (patient group C) have all spoken with the nutritional and healthcare assistant about weight loss. More than two thirds of the respondents have eaten less than usual for short or long periods of time (Table 3a). Many gave as a reason that they have less or no appetite. A number of patients have had diarrhoea, emesis, pain or low spirits, which have caused them to eat scantily or not at all. Many patients in groups A and B express that they *"don't feel like eating the food. If I had other choices, I would eat more"*. Some patients state that they do not have enough time at meal times: *"Everything has to go so fast at meal times – I can't keep up"*.

**Table 3:** Questions and distribution of quantitative answers

Questions and answers\Patient group	A n = 30	B n = 25	C n = 20
a) Have there been days during your stay when you have not eaten much?			
Yes/No/Don't know	24/6/0	17/7/1	17/3/0
b) If yes, has the staff done anything to ensure that you have something to eat and drink?			
Yes/No/Don't know	12/9/3	6/6/5	11/3/3
c) Has the staff given you the impression that food is important for your health?			
Yes/No/Don't know	18/11/1	7/16/2	14/3/3
d) Do you believe food is important for the speed of your recovery?			
Yes/No/Don't know	25/3/2	19/5/1	16/2/1

Before and during the intervention the following patients answer the questions; Group A: patients who are admitted before the nutritional and health assistant is employed on the ward. Group B: patients who are admitted during the employment period of the nutritional and healthcare assistant but not receiving her nutritional care. Group C: patients who are receiving nutritional care from the nutritional and healthcare assistant during their entire or part of their stay at the hospital.

Patients with reduced food and fluid intake are asked whether the staff has attempted to ensure that their nutritional intakes are improved. Half of the patients in group A, one third in group B and two thirds in group C answer "yes" to this question (Table 3b). Patients in groups A and B generally answered, *"The staff has asked whether I would like other things", "They give me water"*, and *"They give me protein drinks"*. However, the patients find that the offers made by the nursing staff are insufficient.

Several of these patients have tried themselves to make an effort to eat more, for instance by asking for ice cream or having family members to bring them food. One patient express it as follows, *"I must do something to be able to go home. I asked for a protein drink today – it wasn't cold – but I hope it was fattening"*. Several patients notice this lack of focus on nutrition: one patient finds it *"rather odd for a hospital not to be leading the way, showing us how to eat*. *They have the opportunity"*. Several patients have specific suggestions for the nutritional care, like *"richer food"*, *"food I can chew" *and *"more soup, for instance split pea soup"*.

Patients in group C who have had days with reduced nutritional intake experience that the nutritional and healthcare assistant takes special care of them: *"It seems to me she does everything"*. *"She comes in later and offers me a second serving. Half an hour before the meal, she brings me an anti-nausea tablet"*. *"She takes special care of us. I missed her at the weekend"*. The patients find that *"the food looks more delicious when the portions are smaller. It makes it more palatable – otherwise you give up from the start"*. The nutritional and healthcare assistant is also reported to be *"good at enticing me: she keeps coming back, reminding me how important proteins are. I feel that she's genuinely interested in each individual person's need for food"*. Patients in group C are also aware that the nutritional and healthcare assistant and the staff enter into records what and how much they eat and drink, and *"if I have eaten or drunk too little, she discusses it with me"*. The knowledge acquired by the patients through their dialogue with the nutritional and healthcare assistant is translated directly into action. For instance the patients deliberately chose certain kinds of foods after a conversation with the staff: "*I have been told a lot about how I should eat more so my weight will be OK and I can get out of here" *and *"She tells me that milk is better for me than fruit juice and that those mashed potatoes are better than boiled potatoes"*. The data suggest that there is a difference between the contents of the patients' dialogue about nutrition with the staff and the contents of their dialogue with the nutritional and healthcare assistant. An example is that the nutritional and healthcare assistant gives the patients specific advice on how to get optimum nutrition.

#### The importance of nutrition for health

The staff has communicated the importance of food to health to two thirds of the patients in group A, in group B one third and more than two thirds in group C (Table 3c). In groups A and B the patients express that the staff keeps telling them to eat and drink because *"if you want to get better you need to eat"*, and the nursing staff often tells them, *"You must eat now"*. Some patients feel pressured to eat, since *"if you reject the food they insist"*. Some patients in group B have been given the impression that food is important for their recovery because of *"the way fellow patients are treated by the nutritional and healthcare assistant"*; *"It is clearly an inspiration to everyone on the ward that the nutritional and healthcare assistant comes here. It makes us think about what we eat, too"*. In group C some patients say more specifically that *"the nutritional and healthcare assistant has discussed with me my lack of strength and has told me I need to eat more"*.

Several patients have noticed that the nutritional and healthcare assistant and the other staff do not always share the same view of the importance of food for the care and treatment: *"the nutritional and healthcare assistant says that food is important, but the others only say that it's important to drink plenty"*. One patient has noticed a difference between the nutritional cares on day and night duty: *"Only the nutritional and healthcare assistant cares about it – the others don't really care. In particular, the staff on evening duty is really tired of keeping dietary records: they say, 'oh, what a load of rubbish"'*.

The majority of all respondents believe that food is important for the speed of their recovery (Table 3d). Many patients express that they know that food is crucial for their recovery: *"I realise that if I don't eat and drink, I won't make it"*. Some patients notice that food also has much impact on their mood.

### Evaluation by the staff

#### Evaluation by the nutritional and healthcare assistant

It is a demanding task for the nutritional and healthcare assistants to build acceptance among their colleagues and to establish a new form of cooperation with a focus on providing nutritional care and managing resources in the nutritional care. The assistants find that their job function require significant dedication, good interpersonal skills, and a strong will to create room for themselves to fulfil their job function.

The assistants find that nutritional care for the patient is the easiest task as *"the patients have been very open and receptive to my advice"*. The very contact with the patients is the best part of the new work function, because *"it's nice to have the possibility and the time to provide this sort of care. Normally, you simply don't have the time for it when you're running around cleaning and doing all sorts of other things"*.

The number of inpatients receiving nutritional care from the nutritional and healthcare assistants varies between the three wards: on one ward 10 to 12 patients will usually receive help whereas on the other two wards the number will be four to eight patients. The assistants organise their daily work in accordance with the number of patients and the patients' condition. The assistants are generally very busy, attending to the nutritional care of six to eight patients. As a result of their work they experince that the majority of the patients at nutritional risk gain weight. The assistants receive mush positive feedback from patients expressing their gratitude for the care the assistants have provided.

#### Division of responsibilities and tasks in the nutritional care

The nursing staff on the three participating wards express that they have accepted the fact that the nutritional and healthcare assistants attend only to nursing tasks related to the nutritional care. For example, the nutritional and healthcare assistants do not respond to patient call bells, assist patients to the toilet or wash patients. Given that the nutritional and healthcare assistants have taken over the nutritional care of the weakest patients, the nursing staff finds that they have more time for their nursing work.

According to the nursing staff, nutritional care tasks are in fierce competition with other nursing tasks for time and, sometimes, interest. As a result, nutritional care tasks are given a low priority. Very often no nursing team member wishes to take over the responsibility of the nutritional and healthcare assistant. Some nutritional care tasks, like keeping dietary records for patients who are nibblers, or recording 24-hour energy and protein intake of patients, are regarded by several nursing team members as boring work, which means that data in the dietary records will be missing or that there will be no continuity, and no decisions are made as to actions that can and shall be taken [32]. Similarly, it is difficult to ensure that the nutritional initiatives implemented and carried out by the nutritional and healthcare assistant on day duty are continued during evening and weekend duty.

The nutritional and healthcare assistants and some senior staff nurses state that the senior staff nurse plays a key role in the successful implementation of the new work function, and that they need her support to further acceptance of the nutritional care and to place it on the ward agenda.

#### Cooperation in the nutritional care

The qualifications, dedication and knowledge of the nutritional and healthcare assistant are appreciated by the majority of the colleagues: *"it's very obvious that after she started in that project everyone pays a lot of attention to the patients' diet and we discuss it more"*. A nurse elaborates, *"I suppose we used to see eating as a necessity and never really thought about it as something that could improve their *[the patients'] *stay here. Meal times have always simply been something to be dispensed with quickly"*. For the nursing staff, and particularly for the newly employed, she serves as a resource person in the nutritional care; she knows the choices offered by the production kitchen and knows how to use these choices to meet patients' individual food requests. Many nursing team members are relieved that they no longer feel guilty about not giving patients sufficient nutrition, because they know that the nutritional and healthcare assistant on day duty will attend to the nibblers' nutritional care.

The nutritional and healthcare assistant reminds the staff to discuss, plan and carry out nutritional tasks in the nursing care; *"Now action is taken much sooner whenever it's clear that things *[nutritional care] *are going in the wrong direction – because she *[the nutritional and healthcare assistant] *is there to point it out to us. If we were to be responsible for noticing it ourselves it might take longer"*. The assistant's attendance at morning conferences may strengthen the focus on nutritional issues and tasks and involve other nursing and medical staff. Thus, through her presence, knowledge and dedication the nutritional and healthcare assistant brings attention to the patients' nutritional care – and to the issue of overweight patients, too, because *"you have a tendency to do nothing, which is wrong, really. For, of course, patients loose the wrong things when they're in here. And that's when she steps in and tells us what we should do"*.

The work of the nutritional and healthcare assistant make visible unnecessary tasks in the nutritional care, like for instance continuous dietary records that are not used in the nutritional care after all. The nursing staff has the experience that "*the patients feel taken special care of" *by the nutritional and healthcare assistant. For instance, *"when she bakes and they can smell fresh bread or cake, it gives them something to look forward to*.

#### Opposition to the organisation model

A few nurses (14% of the senior staff nurses, registered nurses and social and healthcare helpers and assistants) show no appreciation of the importance of nutrition for the care and treatment of the patients, nor do they appreciate the fact that agreements to provide the nutritional care according to the accreditation requirements shall be observed. They are sceptical about the separation of the nutritional care of undernourished patients from the nursing care.

In general, the nursing teams' experience that the nutritional care on evening and weekend duty is going *"downhill" *as compared with day duty, as *"it's the fundamental things that are dealt with: washing and changing – whatever's urgent at the time"*. Most staff members find that on evening and weekend duty *"placing a tray in front of the patient is no problem. But if you're supposed to give them special treatment – well, you can't. The most important thing is that they're washed and wear clean clothes, and that they get food – that is, if they get any. But, at least, you put a tray in front of them ..."*.

#### The ordering of food

Before the intervention, food is usually ordered from the production kitchen by a secretary, who orders a number of portions equivalent to the number of inpatients, but *"no one focused on what we needed on the ward to enable patients to get what they need"*. The nutritional and healthcare assistants' job function is to adjust the food orders to patients' wishes, needs and consumption of food and drink. As a result, according to the senior staff nurses, *"the new system ensures the ward gets the right things *[food and drinks]*"*. A nurse points out that *there's always been this wide choice; we just haven't used it that effectively"*. After the employment of the nutritional and healthcare assistant, the nursing staff has *"realised that there are many options of special diets: vegetarian diet, soft diet, and the patient's foods of choice – I didn't know there were that many options. Before, we offered them something. If the patient doesn't like it? Then what? Should we ring the kitchen or what should we do?"*.

The nursing staff accepts the fact that the intervention involves control of the consumption of food supplies, because *"it's quite nice that she sees to it that the standard is kept. All of the things prescribed by the accreditation, she knows all about that. That's the sort of thing you might usually compromise on, you know"*. For the nutritional and healthcare assistants, on the other hand, it is no easy task to *"come here and change routines. Like, for instance they weren't allowed to eat leftovers. I still sense that some staff members think all this is really odd"*. According to the assistants, it is unpleasant *"hitting barriers from colleagues" *in their work to optimise the use of food supply resources.

#### The perspective of the staff in the production kitchen

In the changed organisation, the staff in the production kitchen communicates directly with the nutritional and healthcare assistants who are responsible for the nutritional care and the ordering of food on the ward and who knows the patients well and the products and services from the production kitchen. The manager in the production kitchen and his staff regard the nutritional and healthcare assistant as their *"ambassador" *on the ward. This is expressed by the manager in the following way: *"It is essential that someone takes the patients' food seriously and that someone cares about their diet so that they'll get what they like. There's no point in us calculating that they *[the patients] *can have 9,000 kJ a day if that's not what they want to eat!"*. When the production kitchen has no such contact, the staff in the kitchen has difficulties getting feedback from the patients and making the wards aware of and use the range of products they offered.

### The amount of food ordered

Table 4 shows key figures during the control and intervention periods of food supply expenses and the total number of inpatients (bed days) on the three participating wards. The average food supply expenses per patient per day amount to EURO 7.0 during the control period and EURO 5.5 during the intervention period. The food supply expenses of the wards decrease by 17 to 27% (20% on average) during the intervention compared with the control period.

**Table 4:** Food supply expenses of the participating wards

	Control period			Intervention		
Category\Ward	I	II	III	I	II	III

Monthly food consumption, EURO	7188	2840	4014	3709	4529	3606
Monthly number of bed days	1,168	367	578	726	726	718
Expenses per bed day, EURO	6.16	7.85	6.95	5.11	6.24	5.03
Change in expenses, %,				- 17	- 17	- 27
Expenses per bed day on average, EURO	7.0			5.5		

Food supply expenses of participating wards I, II and III before and during the intervention. The control period before the intervention data is an average of October and November 2003. During the intervention data is an average of October and November 2004. Source: Data printout from the Office of Economic Affairs and Production Kitchen, Bispebjerg Hospital.

The food and drink ordering pattern changes during the intervention; hence variety in orders increase, for instance more types of bread or cold cuts are ordered, and a larger number of various foods is ordered, like desserts, soups, stewed fruit, bread and cold cuts.

Data from the Office of Financial Affairs at Bispebjerg Hospital (Table 5) show that in the calendar year before the intervention, the three participating wards experience a 10 to 24% increase in food supplies (20% on average). This increase in food supply changes 6 to 10% (7.6% on average) during the first 11 months of the calendar year of the intervention. The fact that the increase in food supply is nearly halved on an annual basis can be explained by the six-month intervention period. These data are supported by production kitchen statements (Table 4). In overall terms, these results suggest that the work of the nutritional and healthcare assistants reduce food supply expenses per patient.

**Table 5:** Changes in food ordered for participating wards

	Food budget*	Food ordered	Increase in food ordered or surplus	Increase in food ordered or surplus
Ward, year	EURO			%

I, 2003 (control)	27069	33200	6131	+22.7
I, 1 Jan – 30 Nov 2004	24085	22738	-1347	-5.6
II, 2003 (control)	16825	18484	1659	+9.9
II, 1 Jan – 30 Nov 2004	26302	24584	-1718	-6.5
III, 2003 (control)	25717	31864	6147	+23.9
III, 1 Jan – 30 Nov 2004	29274	26301	-2973	-10.2

* Ward budgets are prepared on the basis of the number of inpatients per day recorded in the hospital's computer system, the number of inpatients being calculated at the beginning of each day.

Food budget, food ordered, and changes in food ordered for participating wards I, II and III before and with intervention. The control period is the calendar year 2003. The following year includes the intervention period (from 1 July to 31 December 2004). The collecting of data is done one month before termination of the intervention, as the effect is assumed to be at its peak at this time. The increase in consumption has been calculated as a percentage, "+" meaning increase in consumption and "-" meaning surplus. (Source: Data printout from the Office of Economic Affairs, Bispebjerg Hospital)

As a further control, the data are compared with data from the four non-participating wards at the Medical Centre, Bispebjerg Hospital. These four wards experience a 19.3% increase in food supply in relation to the food budget (Table 6). This corresponds with the average 20% increase in food supply of the three participating wards in the year before the intervention (Table 5).

**Table 6:** Food ordered for the non-participating wards

	Food budget*	Food ordered	Increase in food ordered	Increase in food ordered
Ward	EURO			%

IV	30 099	33 059	2 960	+9.8
V	90 077	108 489	18 412	+20.4
VI	71 435	81 681	10 246	+14.3
VII	70 387	89 222	18 835	+26.8

In total	261 998	312 451	50 453	+19.3

* Ward budgets are prepared on the basis of the number of inpatients per day recorded in the hospital's computer system, the number of inpatients being calculated at the beginning of each day.

Food budget, food ordered, and increases in food ordered for the non-participating wards IV-VII at the Medical Centre in 2004. Increase in food ordered is "+"; surplus is "-". (Source: Data printout from the Office of Economic Affairs, Bispebjerg Hospital).

### The amount of food waste

The food waste is calculated as a percentage for four meal types for one control period before the intervention and during the intervention. Towards the end of the intervention, the relative amount of food wastage is approximately reduced by two thirds (specified in Table 7).

**Table 7:** Food wastage at the participating wards

	Control	Intervention		
		
		Beginning	Middle	End
	Wastage rate,%			

Warm breakfast*	49	56	36	17
Lunch	55	37	24	20
Supper	60	34	17	19
"Late evening"**	Not recorded	68	31	17

Average wastage for all meals recorded	55	49	27	18

* Porridge etc.

** Meal served at 8 pm; includes small dishes such as soup, milk beverages, porridge, stewed fruit and cocoa.

Average food wastage at the participating wards I, II and III before and during the employment of the nutritional and healthcare assistant. The control data are collected on three weekdays. The data from the intervention period are collected on a daily basis by the nutritional and healthcare assistant, and represent for 10 days during start-up of the job function (beginning) and after 3 months' (middle) and 5 months' (end) intervention.

## Discussion

Data from interviews with patients not being nursed by the nutritional and healthcare assistants show that the nursing staff often does not talk to the patients about their reduced food and fluid intake and weight loss. The patients, who all can be characterised as nibblers, do not experience that they have a dialogue with the nursing staff about nutritional issues, nor that they are guided in what to eat and drink in order to recover. The patients generally experience that the nutritional care is not a priority in the nursing care; a finding that is supported by similar studies [5,27,28,36]. Most of the patients find themselves that nutrition is very important for their convalescence.

Before the intervention, few patients have experienced any interest in nutritional care on the part of the nursing staff. But during the intervention study the nutritional and healthcare assistants have close communication with the patients and strengthened the focus on preventing undernourishment and weight loss. Patients who are at nutritional risk experience a considerable improvement in the quality of the nursing care in this respect: the assistant ensures that the patient's energy and protein requirements are met by serving small and appetising portions, choosing appropriate foods and keeping the patient focused on the importance of nutrition for his or her recovery. Through this care she imparts knowledge of optimum nutrition to the patient – knowledge that the patient and his/hers family can use after discharge. The patient's own focus on the importance of nutrition for his/hers recovery and efforts to eat sufficiently is likely to be strengthened when a professionally trained person provides care and shows interest in and dedication to nutritional issues [37]. Patients express sincere gratitude, as they have often themselves tried to deal with the problem of reduced appetite and underweight.

All relevant professional groups on the wards experience the job function of the nutritional and healthcare assistant as meaningful. Many nursing team members express their relief that they no longer feel guilty for not providing nibblers with sufficient nutrition. The knowledge and dedication of the nutritional and healthcare assistant carries over to – and strengthened the focus on – the role of nutrition in the nursing care and treatment. Her work renders visible how resources can be used more effectively and how continuity can be established in the nutritional care. The factors that inhibit optimal nutritional care are eliminated. Such factors include lack of time and interest in nutritional care, and that the nursing staff is unable to provide food outside the fixed mealtimes [23,26]. Both patients and staff members express that the intervention is most successful on the day shift, when the nutritional and healthcare assistant is present. This result points out the importance of having a specific staff to provide optimal nutritional care.

A few nurses express a fear that the nutritional care is being separated from the nursing care. They believe that nurses have the required knowledge to attend to this part of the nursing care. Nevertheless, training in nutritional care is often given a low priority in the Danish nursing education [26]. The job function of the nutritional and healthcare assistants may redress the imbalance in daily practice of nutritional care in relation to more technical and medical fields of nursing care. The overall results of her job are an improvement in the quality of the nursing care experienced by the patients and optimisation of the food ordering management. This optimisation and a better utilisation of food supplies can, however, cause disagreement between colleagues, as it tinkers with "old routines" in the food supplies management, for instance by making it clear that the food is for the patients. Yet, the opportunity to build a positive cooperation with the rest of the nursing staff is a crucial factor. In this respect, the management and the senior staff nurse are playing important roles in creating room for the work of the assistant.

The management in the production kitchen experience that with the traditional organisation of the nutritional care, the wards do not have the required competencies, knowledge and work effort to utilise optimally the products offered by the production kitchen. The nutritional and healthcare assistant is as a desired partner ensuring a direct contact between the patients at the ward and the production kitchen.

Before the intervention, the amount of ordered food is based on the number of inpatients. During the intervention, the ordered food is adjusted according to the patients' wishes and requirements. This change produces a surplus of 20% compared with the food budget despite the fact that the nutritional and healthcare assistants order a considerably wider range of food and drink from the production kitchen. The non-participating medical wards at the Medical Centre have a 20% increase in ordered food in relation to the food budget. Besides, the job of the nutritional and healthcare assistants results in a relative reduction in the amount of food wastage from 55% to 18%. Other studies conclude that more than 40% of hospital food is wasted [7,38].

**Figure 1:**
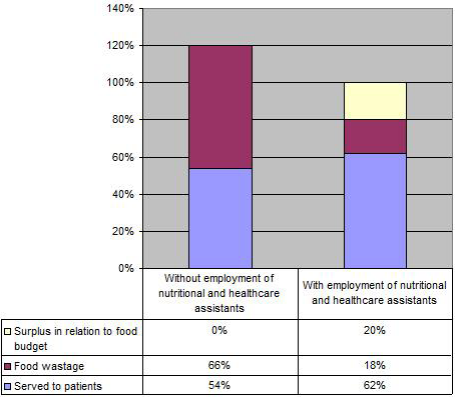
**The use of food supply resources**. The use of food supply resources with and without the employment of nutritional and healthcare assistants. This figure recapitulates Tables 4 to 7. The left-hand column shows how the use of financial resources is distributed on control wards and participating wards before the employment of the nutritional and healthcare assistants. The right-hand column shows how the use of financial resources is distributed on the participating wards after five months' employment of the nutritional and healthcare assistants. The data related to surplus in the food budget are shown in Tables 4 to 6. The data related to the amount of food waste are shown in Table 3. The part of the budget spent on food served to the patients is estimated on the basis of these figures. Plate waste and food eaten by staff members are not taken into account. In the calculations, it is presumed that food waste of the various types of food is weighted equally in terms of expenses. The employment of the nutritional and healthcare assistants results in a surplus of 20% in relation to the food budget and 18% food wastage. Without the assistants the amount in ordered food increases about 20% with 66% food wastage.

The data related to the resources used in the food supply are compared in Figure 1; Before the intervention there is a 20% increase in food ordered (120% in total), 66% of which ends up as food waste. The remaining part of the food budget (54%) is presumed to have been served to patients (Plate wastage and food eaten by staff members are not taken into account). During the last part of the intervention period, 18% of the food budget (100% in total) ends up as food waste, producing a 20% surplus in relation to the food budget. The employment of the nutritional and healthcare assistants substantially optimise food supply resources: food waste is reduced by two thirds, and the food budget is kept – even with a surplus of 20%.

Nutritional care is given a low priority in the hospital sector, the purchase of raw products accounting for no more than a few per cent of the total hospitalisation expenses [26]. Considering the fact that raw materials in the form of food and drink are a vital fuel enabling the patients to combat disease and recover, it is crucial that sufficient financial resources are used for the purchase of raw products for patient food supplies. The financial results obtained with this model support the assumption that there is a significant potential for optimising the use of resources in the nutritional care. Other studies suggest that a reorganisation of the nutritional care is required, with certain staff members being in charge of the nutritional care, if the optimisation of resources is to produce a positive result [39]. Hence, the results from this intervention study provide evidence that the present organisational model is usable. Further investigation at a larger scale is recommended to evaluate the benefit of this organisation of the nutritional care in relation to length of stay, complication rate and readmission.

## Conclusion

Retrained social and healthcare assistants working on wards as nutritional and healthcare assistants strengthen the focus on and raise significantly the quality of the nutritional care of under-resourced patients who are at nutritional risk. Patients give great value to the work of the nutritional and healthcare assistant, as she provides nutritional care to each individual patient and offers them guidance and support in their struggle against weight loss. In contrast, inpatients not attended to by a nutritional and healthcare assistant experience that nutrition is not a priority in the nursing care.

The dedication, knowledge and comprehensive view of the nutritional and healthcare assistants have a positive influence on how their colleagues view and work with the nutritional care. The nutritional and healthcare assistants have a comprehensive view of food orders and adjust them according to the wishes and requirements of the patients. As a result, the food supply resources are used more effectively. The relative amount of food wastage is reduced by two thirds.

The general picture of Danish hospital wards is that no specialists are employed with the same range of competence and field of work as the nutritional and healthcare assistants. In Danish hospitals, quality enhancement and quality measurement are in focus. Hence, the implementation of this organisational model, which is innovative in its field, can be used with great advantage; it has been documented that it raises the quality of the nutritional care for the benefit and delight of patients and staff, and optimises the utilisation of resources. A program describing a specific staff to do the tasks in the day, evening and weekend duty is recommended if undernourishment of patients during hospitalisation shall be eliminated.

## Competing interests

The authors declare that they have no competing interests.

## Authors' contributions

KOL carried out research design and fundraising, designed focus group interviews and questionnaire, interviewed patients and staff, collected data related to the use and wastage of food and performed the analysis of this data, drafted the manuscript and is the guarantor of the manuscript. EG carried out research design, fundraising and coordination of organisational communication and implementation, designed focus group interviews, questionnaire and the implementation of the new organisational structure on the wards, and participated in the data analysis and in the discussion of the manuscript as an author of the manuscript. RN carried out research design, fundraising, and coordination of organisational communication, planning and implementation of the employment of the social and healthcare assistants, employment of the nutritional and healthcare assistants, and implementation of the new organisational structure on the wards and participated in the discussion of the manuscript as an author of the manuscript. All authors have read and approved the final manuscript.

## Pre-publication history

The pre-publication history for this paper can be accessed here:


